# Supplementary material for: 13C values of glycolytic amino acids as indicators of carbohydrate utilization in carnivorous fish
Source: PeerJ. 2019 Sep 18;7:e7701. doi: 10.7717/peerj.7701 (PMC6754727; doi:10.7717/peerj.7701)
Supplement: Supplemental Information 1 [file peerj-07-7701-s001.docx]

**Supplementary Information**

**^13^C values of glycolytic amino acids as indicators of carbohydrate utilization in carnivorous fish**

| Supplemental Table S1 | *δ*^13^C values for non-essential amino acids (NEAA) and bulk *δ*^13^C and *δ*^15^N of individual dietry components and compound diet. |
| --- | --- |
| Supplemental Table S2 | *δ*^13^C values for essential amino acid (EAA) of individual dietry components and compound diet. |
| Supplemental Table S3 | ANOVA results comparing Δ^13^C_F-D_ values of NEAA between the control and experimental treatments. |
| Supplemental Table S4 | ANOVA results comparing Δ^13^C_F-D_ values of EAA between the control and experimental treatments. |
| Supplemental Table S5 | Estimted amino acid (AA) composition differences between salmon muscles and experimental diets. |
| Supplemental Figure S1 | GC-C-IRMS chromatogram of a salmon muscle sample. |
| Supplemental Figure S2 | The Δ^13^C_F-D_ values (mean± SD) of AA and bulk sample between the salmon and the compound diet as well as the individual dietary protein ingredients for control treatment (A) and *Palmaria*_add treatment (B). |

Yiming V. Wang, Alex H. L. Wan, Åshild Krogdahl, Mark P. Johnson, Thomas Larsen

**Supplementary Table S1. Non-essential amino acid *δ*^13^C and bulk *δ*^13^C and *δ*^15^N values of individual protein ingredients and composite diet (‰ VPDB) from both control and experimental diet group.** Triplicate of each dietary sample was analyzed (mean ± SD). Note: Non-essential amino acid and bulk *δ*^13^C (mean ± SD) of fish muscle are previously published in (Wang et al. 2018).

|  | **Non-essential amino acids and bulk** | | | | | | | | | | | | | | | | | | | | | | | | | |  |
| --- | --- | --- | --- | --- | --- | --- | --- | --- | --- | --- | --- | --- | --- | --- | --- | --- | --- | --- | --- | --- | --- | --- | --- | --- | --- | --- | --- |
| **Protein Ingredients** | **Ala** | |  | **Asx** | |  | **Glx** | |  | **Gly** | |  | **Pro** | |  | **Ser** |  |  | **Tyr** | |  | **δ^13^C** | | | **δ^15^N** | | |
| Fish meal | -8.9 | ± | 0.8 | -13.0 | ± | 0.4 | -12.4 | ± | 0.9 | -5.2 | ± | 0.4 | -15.1 | ± | 0.9 | -2.7 | ± | 0.5 | -23.9 | ± | 0.3 | -21.1 | ± | 1.5 | 12.0 | ± | 0.0 |
| *Palmaria palmata* | -11.3 | ± | 0.1 | -12.7 | ± | 0.6 | -14.9 | ± | 0.4 | -14.0 | ± | 0.6 | -17.0 | ± | 0.7 | -11.5 | ± | 2.8 | -25.0 | ± | 0.2 | -20.0 | ± | 2.0 | 6.7 | ± | 0.4 |
| Wheat gluten | -16.6 | ± | 0.7 | -23.4 | ± | 0.7 | -23.1 | ± | 0.4 | -12.0 | ± | 0.9 | -27.1 | ± | 0.4 | -13.5 | ± | 1.3 | -31.2 | ± | 0.3 | -28.8 | ± | 0.1 | 4.3 | ± | 0.0 |
| Pea protein | -16.2 | ± | 0.3 | -21.8 | ± | 0.6 | -22.1 | ± | 1.1 | -17.9 | ± | 0.1 | -24.8 | ± | 0.3 | -16.0 | ± | 0.3 | -31.3 | ± | 0.4 | -27.9 | ± | 0.2 | 0.9 | ± | 0.0 |
|  |  |  |  |  |  |  |  |  |  |  |  |  |  |  |  |  |  |  |  |  |  |  |  |  |  |  |  |
| **Composite diet** |  |  |  |  |  |  |  |  |  |  |  |  |  |  |  |  |  |  |  |  |  |  |  |  |  |  |  |
| Control | -10.8 | ± | 0.6 | -17.6 | ± | 0.2 | -18.5 | ± | 0.3 | -6.4 | ± | 0.4 | -21.0 | ± | 0.4 | -8.0 | ± | 0.4 | -26.9 | ± | 0.5 | -22.2 | ± | 0.4 | 7.0 | ± | 0.6 |
| Palmaria_Add | -11.0 | ± | 1.3 | -16.7 | ± | 0.7 | -18.6 | ± | 0.2 | -8.8 | ± | 0.5 | -21.4 | ± | 0.5 | -10.8 | ± | 0.2 | -27.9 | ± | 0.9 | -23.3 | ± | 0.1 | 6.5 | ± | 0.4 |
|  |  |  |  |  |  |  |  |  |  |  |  |  |  |  |  |  |  |  |  |  |  |  |  |  |  |  |  |
| **Fish** |  |  |  |  |  |  |  |  |  |  |  |  |  |  |  |  |  |  |  |  |  |  |  |  |  |  |  |
| Control | -17.4 | ± | 0.5 | -17.9 | ± | 0.5 | -15.3 | ± | 0.2 | -7.4 | ± | 0.2 | -18.3 | ± | 0.1 | -4.0 | ± | 0.1 | -25.9 | ± | 0.1 | -20.2 | ± | 0.1 | 11.3 | ± | 0.4 |
| Palmaria_Add | -15.1 | ± | 0.2 | -16.3 | ± | 0.3 | -15.0 | ± | 0.1 | -6.4 | ± | 0.2 | -18.3 | ± | 0.6 | -4.9 | ± | 0.3 | -25.9 | ± | 0.2 | -20.2 | ± | 0.2 | 11.1 | ± | 0.4 |
|  |  |  |  |  |  |  |  |  |  |  |  |  |  |  |  |  |  |  |  |  |  |  |  |  |  |  |  |

**Supplementary Table S2. Essential amino acid *δ*^13^C values of individual protein ingredients and composite diet (‰, VPDB) from both control and experimental diet group.** Triplicate of each dietary sample was analyzed (mean ± SD). Note: essential amino acid *δ*^13^C (mean ± SD) of fish muscles are prevsiously published in Wang et al., 2018.

|  | | **Essential amino acids** | | | | | | | | | | | | | | | | | | | | | | | | | |  |
| --- | --- | --- | --- | --- | --- | --- | --- | --- | --- | --- | --- | --- | --- | --- | --- | --- | --- | --- | --- | --- | --- | --- | --- | --- | --- | --- | --- | --- |
| **Protein Ingredients** | | **His** | |  | **Ile** | |  | **Leu** | |  | **Lys** | |  | **Met** | |  | **Phe** | |  | **Thr** | |  | **Val** | |  |  |  |  |
| Fishmeal | -16.2 | | ± | 0.1 | -18.2 | ± | 0.9 | -25.4 | ± | 0.7 | -15.0 | ± | 0.3 | -18.5 | ± | 0.6 | -24.4 | ± | 0.7 | -5.4 | ± | 0.3 | -19.6 | ± | 0.1 |  |  |  |
| *Palmaria palmata* | -17.2 | | ± | 1.7 | -20.4 | ± | 1.4 | -25.3 | ± | 1.7 | -14.0 | ± | 0.4 | -16.8 | ± | 0.7 | -25.2 | ± | 0.3 | -10.0 | ± | 0.8 | -19.8 | ± | 0.3 |  |  |  |
| Pea protein | -24.0 | | ± | 0.1 | -28.3 | ± | 0.6 | -34.4 | ± | 0.4 | -22.3 | ± | 0.6 | -28.4 | ± | 0.7 | -31.1 | ± | 0.4 | -13.5 | ± | 0.0 | -29.7 | ± | 0.4 |  |  |  |
| Wheat gluten | -24.7 | | ± | 0.1 | -25.0 | ± | 0.1 | -34.0 | ± | 0.7 | -23.7 | ± | 0.1 | -26.3 | ± | 0.8 | -31.9 | ± | 1.3 | -8.0 | ± | 1.3 | -30.9 | ± | 0.7 |  |  |  |
|  |  | |  |  |  |  |  |  |  |  |  |  |  |  |  |  |  |  |  |  |  |  |  |  |  |  |  |  |
| **Composite diet** |  | |  |  |  |  |  |  |  |  |  |  |  |  |  |  |  |  |  |  |  |  |  |  |  |  |  |  |
| Control | -17.4 | | ± | 0.3 | -20.0 | ± | 0.7 | -27.6 | ± | 0.4 | -17.8 | ± | 0.2 | -22.5 | ± | 0.7 | -27.2 | ± | 0.4 | -7.8 | ± | 0.4 | -23.8 | ± | 0.6 |  |  |  |
| Palmaria_Add | -18.8 | | ± | 0.3 | -20.6 | ± | 0.4 | -28.0 | ± | 0.1 | -17.2 | ± | 0.3 | -22.3 | ± | 1.0 | -27.4 | ± | 0.5 | -8.0 | ± | 0.2 | -23.7 | ± | 1.0 |  |  |  |
|  |  | |  |  |  |  |  |  |  |  |  |  |  |  |  |  |  |  |  |  |  |  |  |  |  |  |  |  |
| **Fish** |  | |  |  |  |  |  |  |  |  |  |  |  |  |  |  |  |  |  |  |  |  |  |  |  |  |  |  |
| Control | -17.5 | | ± | 0.1 | -19.7 | ± | 0.1 | -27.4 | ± | 0.2 | -17.2 | ± | 0.1 | -21.2 | ± | 0.1 | -27.3 | ± | 0.1 | -7.2 | ± | 0.3 | -23.9 | ± | 0.2 |  |  |  |
| Palmaria_Add | -18.2 | | ± | 0.1 | -20.2 | ± | 0.3 | -27.8 | ± | 0.1 | -17.2 | ± | 0.2 | -22.5 | ± | 0.0 | -27.7 | ± | 0.3 | -7.2 | ± | 0.3 | -23.7 | ± | 0.0 |  |  |  |

**Supplemental Table S3. ANOVA results comparing NEAA Δ13C_F-D_ values between the control and experimental treatments.**

**Ser** :

**Df Sum Sq Mean Sq F value P-adjusted**

Regression 1 5.0417 5.0417 100.6 0.0019 **

Residuals 4 0.2005 0.0501

**Gly** :

**Df Sum Sq Mean Sq F value P-adjusted**

Regression 1 17.1704 17.1704 550.04 1.371e-04 ***

Residuals 4 0.1249 0.0312

**Ala** :

**Df Sum Sq Mean Sq F value P-adjusted**

Regression 1 10.1140 10.1140 82.017 0.0019 **

Residuals 4 0.4933 0.1233

**Glx** :

**Df Sum Sq Mean Sq F value P-adjusted**

Regression 1 0.33607 0.33607 12.602 0.0333

Residuals 4 0.10667 0.02667

**Asx** :

**Df Sum Sq Mean Sq F value P-adjusted**

Regression 1 0.57042 0.57042 3.58 0.1533

Residuals 4 0.63733 0.15933

**Pro** :

**Df Sum Sq Mean Sq F value P-adjusted**

Regression 1 0.32667 0.32667 1.8101 0.2497

Residuals 4 0.72187 0.18047

**Tyr** :

**Df Sum Sq Mean Sq F value P-adjusted**

Regression 1 1.64327 1.64327 58.341 0.0027 **

Residuals 4 0.11267 0.02817

**Supplementary Table S4. ANOVA results comparing EAA Δ13C_F-D_ values between the control and experimental treatments.**

Thr :

**Df Sum Sq Mean Sq F value P-adjusted**

Regression 1 0.058017 0.058017 0.7432 0.700

Residuals 4 0.312267 0.078067

Lys :

**Df Sum Sq Mean Sq F value P-adjusted**

Regression 1 0.82140 0.82140 27.719 0.0166

Residuals 4 0.11853 0.02963

His :

**Df Sum Sq Mean Sq F value P-adjusted**

Regression 1 0.62727 0.62727 74.379 0.0039 **

Residuals 4 0.03373 0.00843

Ile :

**Df Sum Sq Mean Sq F value P-adjusted**

Regression 1 0.000267 0.000267 0.0041 0.9517

Residuals 4 0.257267 0.064317

Met :

**Df Sum Sq Mean Sq F value P-adjusted**

Regression 1 3.13927 3.13927 1088.8 4.024e-05 ***

Residuals 4 0.01153 0.00288

Val :

**Df Sum Sq Mean Sq F value P-adjusted**

Regression 1 0.032267 0.032267 2.3269 0.4036

Residuals 4 0.055467 0.013867

Phe :

**Df Sum Sq Mean Sq F value P-adjusted**

Regression 1 0.014017 0.014017 0.2775 0.8349

Residuals 4 0.202067 0.050517

Leu :

**Df Sum Sq Mean Sq F value P-adjusted**

Regression 1 0.001067 0.0010667 0.0593 0.9367

Residuals 4 0.071933 0.0179833

**Supplementary Table S5. Estimted amino acid (AA) composition differences between of salmon muscles and experimental diets.**

Salmon AA composition data is based on (Wilson & Cowey 1985)*. The Estimated AA composition of the compound diets are calculated from protein concentration weighted AA composition of each protein ingredients. The AA compositions from individual protein source are based on previsouly published AA composition of fishmeal (FAO 2017), wheat gluten (Rombouts et al. 2009), pea protein (Leterme et al. 1990) and *Palmaria palmate* (Mæhre et al. 2014). Note that for the estimated molar balance, 100% means identical molar composition; <100% means dietary deficiency for a given AA, and vice versa for > 00%.

| **Control experiment** | **Ser** | **Gly** | **Ala** | **Glx** | **Asx** | **Pro** | **Tyr** |  | **Thr** | **Lys** | **His** | **Ile** | **Met** | **Phe** | **Leu** | **Val** |
| --- | --- | --- | --- | --- | --- | --- | --- | --- | --- | --- | --- | --- | --- | --- | --- | --- |
| **Control diet**  **(g/100g protein DW)** | 4.22 | 6.38 | 5.32 | 20.01 | 8.29 | 5.60 | 3.07 |  | 3.79 | 6.23 | 2.09 | 4.09 | 2.17 | 4.02 | 6.94 | 4.83 |
| **Salmon muscle**  **(g/100g protein DW)** | 4.61 | 7.41 | 6.52 | 14.30 | 9.92 | 4.64 | 3.50 |  | 4.95 | 9.28 | 3.02 | 4.41 | 1.83 | 4.38 | 7.72 | 5.09 |
| **Estimated molar balance (%)** | 91.5 | 86.1 | 81.6 | 139.9 | 83.6 | 120.7 | 87.7 |  | 76.6 | 67.1 | 69.2 | 92.7 | 118.6 | 91.8 | 89.9 | 94.9 |
|  |  |  |  |  |  |  |  |  |  |  |  |  |  |  |  |  |
|  |  |  |  |  |  |  |  |  |  |  |  |  |  |  |  |  |
|  |  |  |  |  |  |  |  |  |  |  |  |  |  |  |  |  |
| **Palmaria_add experimnet** | **Ser** | **Gly** | **Ala** | **Glx** | **Asx** | **Pro** | **Tyr** |  | **Thr** | **Lys** | **His** | **Ile** | **Met** | **Phe** | **Leu** | **Val** |
| **Palmaria_add Diet**  **(g/100g protein DW)** | 4.36 | 6.28 | 5.51 | 20.20 | 8.34 | 5.78 | 3.12 |  | 3.88 | 6.16 | 2.02 | 3.95 | 2.13 | 4.15 | 6.76 | 4.99 |
| **Salmon muscle**  **(g/100g protein DW)** | 4.61 | 7.41 | 6.52 | 14.30 | 9.92 | 4.64 | 3.50 |  | 4.95 | 9.28 | 3.02 | 4.41 | 1.83 | 4.38 | 7.72 | 5.09 |
| **Estimated molar balance (%)** | 94.6 | 84.8 | 84.5 | 141.3 | 84.1 | 124.6 | 89.1 |  | 78.4 | 66.4 | 66.9 | 89.6 | 116.4 | 94.7 | 87.6 | 98.0 |

* Several studies show that that AA compositions of Atlantic salmon fed on different type of diet are not significantly different given the optimum protein are provided in the diet (Rollin et al. 2006).

Supplemental Figure S1.

**A GC-C-IRMS chromatogram of individual amino acids from the muscle tissue of the Atlantic salmon (*Salmo salar*) where the intensity represents voltage (mV) of ion mass 44.** GC-C-IRMS stands for Gas Chromatograph-Combustion-Isotope Ratio Mass Spectrometry (see methods for instrument specification).


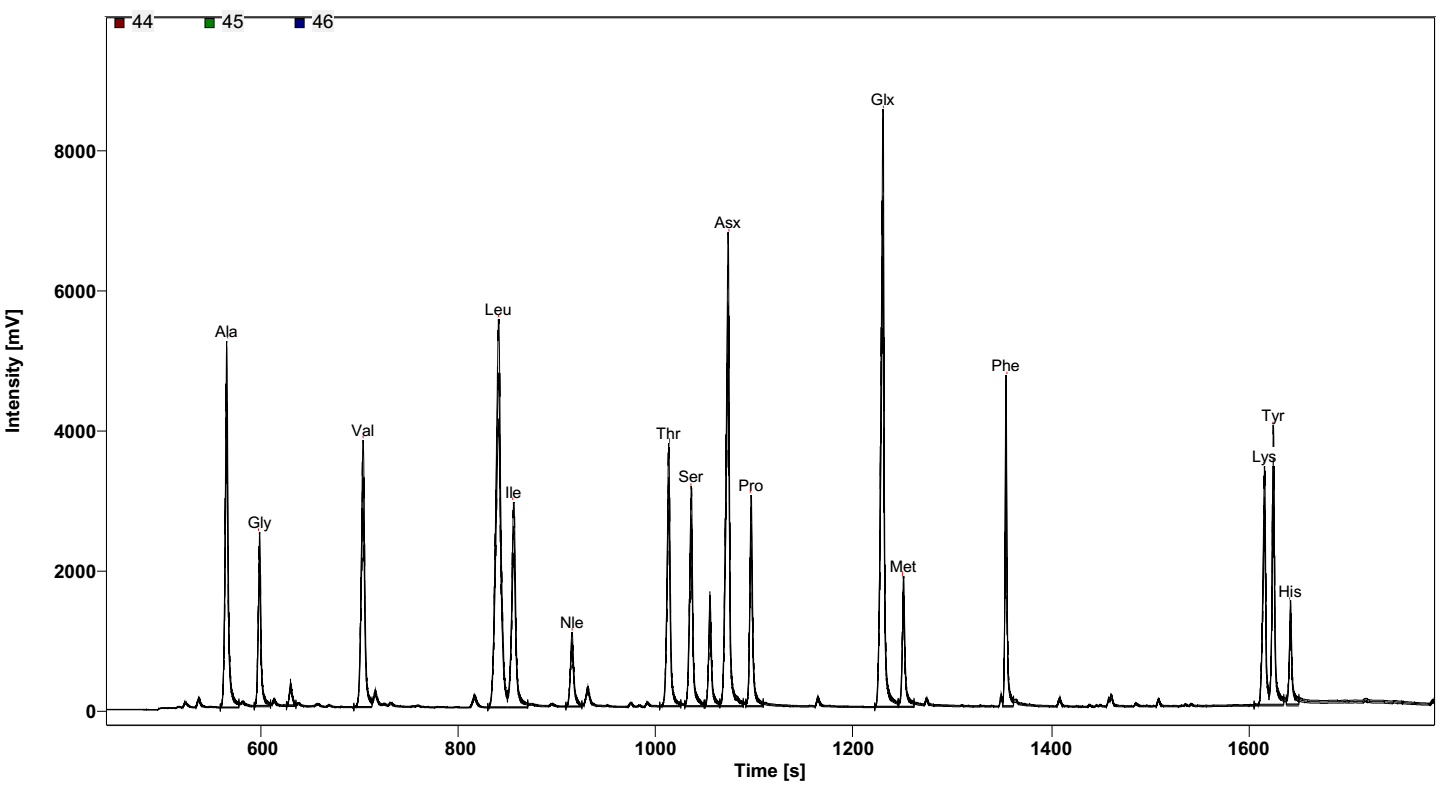


Supplementary Figure S2.

The Δ^13^C_F-D_ values (mean± SD) of AA and bulk sample between the salmon and the compound diet as well as the individual dietary protein ingredients for control treatment (A) and *Palmaria*_add treatment (B).

**Supplemental Reference:**

FAO. 2017. FAO Fisheries and aquaculture - Global Aquaculture Production (FishStatJ).

Leterme P, Monmart T, and Baudart E. 1990. Amino acid composition of pea (Pisum sativum) proteins and protein profile of pea flour. *Journal of the Science of Food and Agriculture* 53:107-110. 10.1002/jsfa.2740530112

Mæhre HK, Malde MK, Eilertsen K-E, and Elvevoll EO. 2014. Characterization of protein, lipid and mineral contents in common Norwegian seaweeds and evaluation of their potential as food and feed. *Journal of the Science of Food and Agriculture* 94:3281-3290. 10.1002/jsfa.6681

Rollin X, Wauters J-B, Lie Bodin N, Larondelle Y, Ooghe W, Wathelet B, and Abboudi T. 2006. *Maintenance threonine requirement and efficiency of its use for accretion of whole-body threonine and protein in Atlantic salmon (Salmo salar L.) fry*.

Rombouts I, Lamberts L, Celus I, Lagrain B, Brijs K, and Delcour JA. 2009. Wheat gluten amino acid composition analysis by high-performance anion-exchange chromatography with integrated pulsed amperometric detection. *Journal of Chromatography A* 1216:5557-5562. <https://doi.org/10.1016/j.chroma.2009.05.066>

Wang YV, Wan AHL, Lock E-J, Andersen N, Winter-Schuh C, and Larsen T. 2018. Know your fish: A novel compound-specific isotope approach for tracing wild and farmed salmon. *Food Chemistry* 256:380-389. <https://doi.org/10.1016/j.foodchem.2018.02.095>

Wilson RP, and Cowey CB. 1985. Amino acid composition of whole body tissue of rainbow trout and Atlantic salmon. *Aquaculture* 48:373-376. <http://dx.doi.org/10.1016/0044-8486(85)90140-1>
